# Canonical information flow decomposition among neural structure subsets

**DOI:** 10.3389/fninf.2014.00049

**Published:** 2014-05-30

**Authors:** Daniel Y. Takahashi, Luiz A. Baccalá, Koichi Sameshima

**Affiliations:** ^1^Psychology Department, Neuroscience Institute, Princeton UniversityPrinceton, NJ, USA; ^2^Telecommunications and Control Department, Escola Politécnica, University of São PauloSão Paulo, Brazil; ^3^Department of Radiology and Oncology, Faculdade de Medicina, University of São PauloSão Paulo, Brazil

**Keywords:** directed connectivity measures, canonical decomposition, frequency domain, information flow, generalized coherence

## Abstract

Partial directed coherence (PDC) and directed coherence (DC) which describe complementary aspects of the directed information flow between pairs of univariate components that belong to a vector of simultaneously observed time series have recently been generalized as bPDC/bDC, respectively, to portray the relationship between subsets of component vectors (Takahashi, 2009; Faes and Nollo, 2013). This generalization is specially important for neuroscience applications as one often wishes to address the link between the set of time series from an observed ROI (region of interest) with respect to series from some other physiologically relevant ROI. bPDC/bDC are limited, however, in that several time series within a given subset may be irrelevant or may even interact opposingly with respect to one another leading to interpretation difficulties. To address this, we propose an alternative measure, termed cPDC/cDC, employing canonical decomposition to reveal the main frequency domain modes of interaction between the vector subsets. We also show bPDC/bDC and cPDC/cDC are related and possess mutual information rate interpretations. Numerical examples and a real data set illustrate the concepts. The present contribution provides what is seemingly the first canonical decomposition of information flow in the frequency domain.

## 1. Introduction

Human behavior is primarily thought as a property that emerges from the interaction of several brain areas, body parts, and the environment. Understanding how these elements dynamically interact is one of major themes of systems neuroscience. Several multivariate time series methods—old and new—have been introduced to describe the interdependence between brain areas using signal modalities like EEG, BOLD signals, MEG and LFP—and are collectively called connectivity measures. *Partial directed coherence* (PDC) (Baccalá and Sameshima, 2001) and *directed coherence/directed transfer function* (DC/DTF) (Kamiński and Blinowska, 1991) are two examples of such connectivity measures. Both describe complementary aspects (see Baccalá and Sameshima, 2014 for an in depth discussion) of how information flows between pairs of univariate time series components that belong to a multivariate vector of simultaneously observed time series (Takahashi et al., 2010). Recently, PDC and DC have been generalized (as bPDC/bDC, respectively) to describe how subsets (blocks) of components within a time series vector interrelate (Takahashi, 2009; Faes and Nollo, 2013). This is specially important for neuroscience applications as one often wants to investigate the interaction between sets of time series that are circumscribed to an observed region of interest (ROI) with respect to another physiologically relevant ROI (Nedungadi et al., 2011). The potential relevance of this type of question alone justifies looking for their deeper meaning in terms of information theoretical quantities.

Despite their practical importance, bPDC/bDC suffer from the limitation that several time series within a given subset may be irrelevant or interact in opposition to one another thereby posing interpretation difficulties. Also, in several situations, a researcher may be interested in just the few “best” descriptions of interaction between two sets of time series but not in the total amount of information flowing between them. For a more concrete example, assume that two brain areas interact and that bPDC is large. In this situation, it does not straightforwardly follow that all brain region components are interacting in the same way, or even whether some such components may be ignored. One way to address this limitation is to decompose bPDC/bDC into different components weighed according to relevance.

The aim of this article is twofold: (a) to provide a proper information theoretic interpretation for bPDC/bDC and (b) to introduce a canonical decomposition of information flows, henceforth termed, respectively, canonical PDC/DC (cPDC/cDC). These new decompositions allow us to closely mimic classical canonical correlation analysis so that different dynamically relevant interaction modes between brain areas can be exposed. Due to PDC interpretability in terms of Granger causality (Baccalá and Sameshima, 2014), a consequence of the present formulation is that cPDC represents a long sought frequency domain counterpart to time domain canonical decompositions of Granger causality (Sato et al., 2010; Ashrafulla et al., 2013).

The article is organized as follows. We first introduce the background and notation necessary for the rest of the article (section 2). In the results section (section 3), we first show that both bPDC and bDC between two subsets of processes are block coherences between suitably defined underlying processes. Then, we demonstrate that such coherences are nothing but monotonic transformations of the mutual information rate between the respective processes (Gelfand and Yaglom, 1959; Takahashi et al., 2010; Nedungadi et al., 2011) leading immediately to their interpretability as mutual information rates. Next, we introduce cPDC and cDC and prove that they are the non-zero eigenvalues of the matrices whose determinants underlie the respective bPDC and bDC definitions (section 4). Using simulated examples and publicly available data we illustrate the usefulness of cPDC/cDC (section 5) followed by a brief discussion (section 6). Proof details are left to the Appendix.

## 2. Background

Let *X*_1_, …, *X_K_* be *K* distinct multivariate time series vectors with dimension *M*_1_, …, *M_K_*. Using ^*T*^ to indicate matrix transposition, let *X*(*t*) = [*X*_1_(*t*)^*T*^, …, *X_K_(t)^T^*]^*T*^ for each time *t* ∈ ℤ be a second order stationary time series with spectral density matrix *S*(ω) at each frequency ω ∈ [−π, π). To justify our formal computation, we assume that *S*(ω) is uniformly bounded from below and above and invertible at all frequencies (Hannan, 1970). This is called the *boundedness* condition which guarantees that the following autoregressive (AR) representation of *X* holds in the mean square sense

(1) $$X(t)=\sum_{l=0}^{+\infty }H(l)\epsilon (t-l),$$

where ϵ(*t*) = [ϵ_1_(*t*)^*T*^ … ϵ*_K_(t)^T^*]*^T^* stands for a zero mean innovation process, i.e., 𝔼[ϵ(*t*)ϵ(*t*)^*T*^] = Σ and 𝔼[ϵ(*t*)ϵ(*l*)^*T*^] = 0 for *l* ≠ *t*. For *l* ≥ 1, *A*(*l*) are (*M*_1_ + … + *M_K_*)^2^-dimensional matrices. Let *A*_*pq*_(*l*) for *p*, *q* ∈ {1, …, *K*} and *l* ≥ 1 be *M*_*p*_ × *M_q_*-dimensional matrices so that *A*(*l*) has the following structure

$$A(l)=\left[\begin{array}{ccc} A_{11}(l) & \ldots & A_{1M}(l)\\ \vdots & \ddots & \vdots \\ A_{M1}(l) & \ldots & A_{MM}(l) \end{array}\right]$$

We define $\bar{A}(\omega )=I-\sum_{l\geq 1}A(l)e^{-\sqrt{-1}\omega l}$.

Under the boundedness condition, the following moving average (MA) mean square sense representation for the process *X* also holds

(2) $$X(t)=\sum_{l=0}^{+\infty }H(l)\epsilon (t-l),$$

where *H*(*l*) for *l* ≥ 0 are (*M*_1_ + … + *M_K_*)^2^-dimensional matrices. Let $\bar{H}(\omega )=\sum_{l\geq 0}H(l)e^{-\sqrt{-1}\omega l}$. We have that Ā^*^(ω) = *H*^−1^(ω) for all ω ∈ [−π, π). The superscript * indicates the matrix complex conjugate.

Let *P*(ω) = *S*^−1^(ω). bPDC from the multivariate process *X*_*j*_ to the process *X*_*i*_ at frequency ω, denoted π^(*b*)^_*ij*_(ω), is defined (Takahashi, 2009; Faes and Nollo, 2013) by

(3) $$\pi_{ij}^{\left(b\right)}(\omega )=1-\det\text{​}\left(P_{jj}(\omega )-{\bar{A}}_{ij}^{*}(\omega )\Sigma_{ii}^{-1}{\bar{A}}_{ij}(\omega )\right)\det\text{​}{\left(P_{jj}(\omega )\right)}^{-1},$$

where det indicates the determinant and the subscript indices relate to the natural block structure associated with the matrices.

Let Θ = Σ^−1^. bDC from the multivariate process *X*_*j*_ to the process *X*_*i*_ at frequency ω, denoted γ^(*b*)^_*ij*_(ω), is defined (Takahashi, 2009; Faes and Nollo, 2013) by

(4) $$\gamma_{ij}^{\left(b\right)}(\omega )=1-\det\text{​}\left(S_{ii}(\omega )-{\bar{H}}_{ij}(\omega )\Theta_{jj}^{-1}{\bar{H}}_{ij}^{*}(\omega )\right)\det{\left(S_{ii}(\omega )\right)}^{-1}.$$

Note that the present bDC definition differs slightly from the one in Faes and Nollo (2013). We removed the unnecessary condition of strict causality, i.e., diagonality of Σ, simply by substituting Σ^−1^_*jj*_ by Θ^−1^_*jj*_ in their definition of bDC as it is more suited for formulating information theoretic results as shown ahead.

Consider a second-order stationary multivariate process *W*(*t*) = [*Y(t)^T^ Z(t)^T^*]^*T*^. The block coherence between *Y* and *Z* at frequency ω is defined as (Nedungadi et al., 2011)

(5) $$C_{YZ}^{\left(b\right)}(\omega )=1-\det\left(S_{WW}(\omega )\right)\det{\left(S_{YY}(\omega )\right)}^{-1}\det{\left(S_{ZZ}(\omega )\right)}^{-1}\text{​​}.$$

Observe that we used the process name in the subscript of the power spectrum *S* to indicate the corresponding spectral density matrices. In the rest of the article, we will use interchangeably the process name or the corresponding indices in the subscript whenever there is no ambiguity.

Another important definition is that of mutual information rate (MIR) between two multivariate strictly stationary processes *Y* and *Z* is

(6) $${\text{MIR}}_{YZ}=\underset{t\to +\infty }{\lim}\frac{1}{t}𝔼\left[\log\frac{d\mathbb{P}(Y(1),\ldots ,Y(t),Z(1),\ldots ,Z(t))}{d\mathbb{P}(Y(1),\ldots ,Y(t))d\mathbb{P}(Z(1),\ldots ,Z(t))}\right]\text{​}.$$

The classical relationship between block coherence (Equation 5) and mutual information rate (Equation 6) follows from

**Theorem**. (*Gelfand and Yaglom, 1959; Pinsker, 1964) If Y and Z are jointly stationary Gaussian processes satisfying the boundedness condition, we have that the MIR between Y and Z is given by*

(7) $${\text{MIR}}_{YZ}=-\frac{1}{4\pi }\int_{-\pi }^{\pi }\log\left(1-C_{YZ}^{\left(b\right)}(\omega )\right)d\omega .$$

Now, following Takahashi et al. (2010), we define, for *i* ∈ {1, …, *K*}, the partialized process η_*i*_ by

(8) $$\eta_{i}(t)=X_{i}(t)-\;𝔼\left[X_{i}(t)|\left\{X_{j}(l),j\neq i,l\in \mathbb{Z}\right\}\right],$$

where 𝔼[⊙|⊙] henceforth denotes the best linear conditional predictor. Likewise the partialized innovation process ζ_*i*_ for *i* ∈ {1, …, *K*} is

(9) $$\zeta_{i}(t)=\epsilon_{i}(t)-\;𝔼\left[\epsilon_{i}(t)|\left\{\epsilon_{j}(t),j\neq i\right\}\right].$$

Observe that both partialized process and partialized innovation process were defined in Takahashi et al. (2010) but for the special case of scalar η_*i*_ and ζ_*i*_.

## 3. Relation between bPDC/bDC and mutual information rate

Our first result establishes the relationship between bPDC and block coherence and is analogous to Theorem 1 in Takahashi et al. (2010).

**Theorem 1**. *Let X satisfy the boundedness condition. For all i, j* ∈ {1, …, *K*} *and all frequencies* ω ∈ [−π, π) *we have that*

(10) $$\pi_{ij}^{\left(b\right)}(\omega )=C_{\epsilon_{i}\eta_{j}}^{\left(b\right)}(\omega ).$$

A straightforward corollary is

**Corollary 1**. *Let X be a stationary Gaussian process and satisfy the boundedness condition. For all i, j* ∈ {1, …, *K*} *we have that*

(11) $${\text{MIR}}_{\epsilon_{i}\eta_{j}}=-\frac{1}{4\pi }\int_{-\pi }^{\pi }\log\left(1-\pi_{ij}^{\left(b\right)}(\omega )\right)d\omega .$$

Similar results also hold for bDC.

**Theorem 2**. *Let X satisfy the boundedness condition. For all i, j* ∈ {1, …, *K*} *and all frequencies* ω ∈ [−π, π) *we have that*

(12) $$\gamma_{ij}^{\left(b\right)}(\omega )=C_{X_{i}\zeta_{j}}^{\left(b\right)}(\omega ).$$

and

**Corollary 2**. *Let X be a stationary Gaussian process and satisfy the boundedness condition. For all i, j* ∈ {1, …, *K*}, *we have that*

(13) $${\text{MIR}}_{X_{i}\zeta_{j}}=-\frac{1}{4\pi }\int_{-\pi }^{\pi }\log\left(1-\gamma_{ij}^{\left(b\right)}(\omega )\right)d\omega .$$

## 4. Canonical PDC and DC

Canonical correlation is a classical method developed initially by Hotelling (1936) to address the relationship between random vectors. Brillinger (1981) generalized the method for time series and gave an excellent account of the relationship between canonical correlation analysis and different ideas in multivariate statistics. Our formulation of canonical coherence is equivalent to the definition introduced by Brillinger (1981).

Let *Y* and *Z* be respectively *M*_*Y*_- and *M*_*Z*_-dimensional jointly second order stationary processes. To better understand the relationship between *Y* and *Z*, we can ask the following question: Which components of *Y* and *Z* are most representative of the interaction between the processes? One way to formalize this is to consider filtering matrices *B_Y_(l)* (1 × *M_Y_*) and *B_Z_(l)* (1 × *M_Z_*), for all *l* ∈ ℤ and define the scalar processes *b*_*Y*_ and *b*_*Z*_ by

(14) $$b_{Y}(t)=\sum_{l\in \mathbb{Z}}B_{Y}(l)Y(t-l)$$

and

(15) $$b_{Z}(t)=\sum_{l\in \mathbb{Z}}B_{Z}(l)Z(t-l),$$

so that *C_b_Y_b_Z__*(ω) is maximized for all ω ∈ [−π, π). If furthermore *Y* and *Z* are jointly stationary Gaussian processes, then this is equivalent to maximizing *MIR_b_Y_b_Z__*.

Following the above idea, we define the first canonical coherence between *Y* and *Z* at frequency ω by

(16) $$C_{YZ}^{\left(c_{1}\right)}(\omega )=\underset{B_{Y},B_{Z}}{\sup}C_{b_{Y}b_{Z}}(\omega ).$$

Assume that the supremum (Equation 16) is achieved for *b*_*Y*_ and *b_Z_*, which we call *first canonical time series*. Consider the residual processes *Y*^1^(*t*) = *Y(t)* − 𝔼[*Y(t)*| {*b_Y_(l)*, *l* ∈ ℤ}] and *Z*^1^(*t*) = *Z(t)* − 𝔼[*Z(t)*| {*b_Z_(l)*, *l* ∈ ℤ}]. Observe that *Y*^1^ and *Z*^1^ are uncorrelated to the processes *b_Y_* and *b_Z_*, respectively. The second canonical coherence *C*^(*c*_2_)^_*YZ*_(ω) is defined recursively on the residues by *C*^(*c*_2_)^_*YZ*_(ω) = *C*^(*c*_1_)^_*Y*^1^*Z*^1^_(ω).

Analogously, for 2 ≤ *m* ≤ min{*M_Y_, M_Z_*}, considering the residual processes

$$\begin{array}{l} Y^{m}(t)=Y^{m-1}(t)-𝔼\text{​}[Y^{m-1}(t)|\{{\bar{b}}_{Y^{k}}(l),l\in \mathbb{Z},\\ \;k\in \{1,\ldots ,m-1\}\}] \end{array}$$

and

$$\begin{array}{l} Z^{m}(t)=Z^{m-1}(t)-𝔼\text{​}[Z^{m-1}(t)|\{{\bar{b}}_{Z^{k}}(l),l\in \mathbb{Z},\\ \;k\in \{1,\ldots ,m-1\}\}]\text{​}, \end{array}$$

one may define the *m*-th canonical coherence as

(17) $$C_{YZ}^{\left(c_{m}\right)}(\omega )=C_{Y^{m-1}Z^{m-1}}^{\left(c_{1}\right)}(\omega ).$$

In this way, it is possible to construct a hierarchy of coherences where each element captures the dependence structure that is not explained by the other elements.

Finally, we introduce cPDC and cDC. For *m* ≤ min{*M_i_, M_j_*}, the **m*-th canonical* PDC from *j* to *i* at frequency ω denoted π^(*c_m_*)^_*ij*_(ω) is defined by

(18) $$\pi_{ij}^{\left(c_{m}\right)}(\omega )=C_{\epsilon_{i}\eta_{j}}^{\left(c_{m}\right)}(\omega ).$$

Similarly, the **m*-th canonical* DC from *j* to *i* at frequency ω denoted γ^(*c_m_*)^_*ij*_(ω) is defined by

(19) $$\gamma_{ij}^{\left(c_{m}\right)}(\omega )=C_{X_{i}\zeta_{j}}^{\left(c_{m}\right)}(\omega ).$$

At first sight, it is unclear whether the canonical PDC and DC exist at all or even if they are uniquely defined. More importantly, nor is it obvious that it is possible to compute them. Despite these initial uncertainties, we show next that canonical coherences are consistently defined as the non-null eigenvalues of some specific matrices.

Let λ^*m*^(*Q*) denote its *m*-th eigenvalue from matrix *Q* ordered from its largest to its smallest value. The following theorem furnishes a practical way to calculate cPDC and cDC.

**Theorem 3**. *Under the boundedness condition for X the following identities hold:*

(20) $$\pi_{ij}^{\left(c_{m}\right)}(\omega )=\lambda^{m}\left({\bar{A}}_{ij}^{*}(\omega )\Sigma_{ii}^{-1}{\bar{A}}_{ij}(\omega )P_{jj}^{-1}(\omega )\right)$$

and

(21) $$\gamma_{ij}^{\left(c_{m}\right)}(\omega )=\lambda^{m}\left(S_{ii}^{-1}(\omega ){\bar{H}}_{ij}(\omega )\Theta_{jj}^{-1}{\bar{H}}_{ij}^{*}(\omega )\right)\text{​}.$$

Furthermore it is possible to relate bPDC/bDC and cPDC/cDC via

**Theorem 4**. *Under the same conditions of Theorem 3 the following identities hold:*

(22) $$\pi_{ij}^{\left(b\right)}(\omega )=1-\prod_{m=1}^{\min\{M_{i},M_{j}\}}\left(1-\pi_{ij}^{\left(c_{m}\right)}(\omega )\right)$$

and

(23) $$\gamma_{ij}^{\left(b\right)}(\omega )=1-\prod_{m=1}^{\min\{M_{i},M_{j}\}}\left(1-\gamma_{ij}^{\left(c_{m}\right)}(\omega )\right)\text{​}.$$

A simple consequence of Equations (22), (23) is that for stationary Gaussian processes satisfying the boundedness condition, we now have a decomposition of the mutual information rates

(24) $${\text{MIR}}_{\epsilon_{i}\eta_{j}}=\sum_{m=1}^{\min\{M_{i},M_{j}\}}-\frac{1}{4\pi }\int_{-\pi }^{\pi }\log\left(1-\pi_{ij}^{\left(c_{m}\right)}(\omega )\right)d\omega$$

and

(25) $${\text{MIR}}_{X_{i}\zeta_{j}}=\sum_{m=1}^{\min\{M_{i},M_{j}\}}-\frac{1}{4\pi }\int_{-\pi }^{\pi }\log\left(1-\gamma_{ij}^{\left(c_{m}\right)}(\omega )\right)d\omega .$$

Note how the quantities being summed in Equations (24), (25) are formally themselves contributions to the mutual information written in terms of their canonical coherence contributions.

## 5. Illustrations

### 5.1. Simulated models

*Example 1*. To provide insight into cPDC behavior, we begin with a very simple example that can be fully and explicitly solved.

Let a vector of observed time series [*Y*_1_, *Y*_2_, *Y*_3_, *Y*_4_] be a real valued autoregressive process of order *p* = 1 and Σ = I. The autoregressive coefficients of the model are described by

(26) $$A(1)=\left(\begin{array}{cccc} .5 & f & 0 & 0\\ e & .5 & 0 & 0\\ a & b & .5 & h\\ c & d & g & .5 \end{array}\right)\text{​},$$

as in Figure 1.

**Figure 1:**
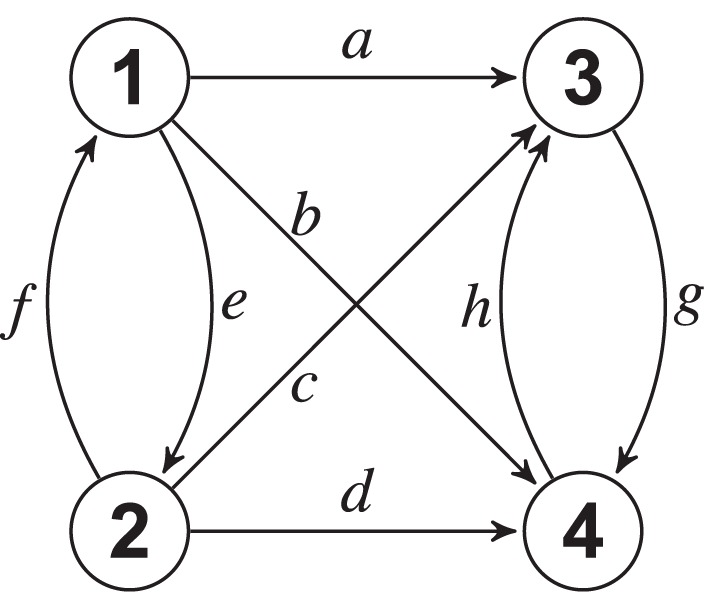
**Connectivity diagram for Example 1**. The number of canonical components depends on the value of *ad* − *bc*.

By adopting time series blocks as *X*_1_ = [*Y*_1_ *Y*_2_] and *X*_2_ = [*Y*_3_ *Y*_4_], when *e* = f = g = h = 0, direct computation shows that the canonical PDC from block *X*_2_ to *X*_1_ is zero, i.e., π^(*c*_1_)^_12_(ω) = π^(*c*_2_)^_12_(ω) = 0 for all ω (reflecting the nullity of the 2 × 2 *A*(*l*) right side upper block), whereas the coupling in the opposite direction contributes two distinct components:

(27) $$\pi_{21}^{\left(c_{1}\right)}(\omega )=\frac{a^{2}+b^{2}+c^{2}+d^{2}+\sqrt{{\left(a^{2}+b^{2}+c^{2}+d^{2}\right)}^{2}-4{\left(ad-bc\right)}^{2}}}{2.5-2\cos(\omega )}$$

and

(28) $$\pi_{21}^{\left(c_{2}\right)}(\omega )=\frac{a^{2}+b^{2}+c^{2}+d^{2}-\sqrt{{\left(a^{2}+b^{2}+c^{2}+d^{2}\right)}^{2}-4{\left(ad-bc\right)}^{2}}}{2.5-2\cos(\omega )}.$$

For *ad* = *bc*—i.e., if the lower left 2× 2 block determinant of *A*(*l*) is zero as well, the total number of non-zero cPDC components reduces to just 1.

**Figure 2:**
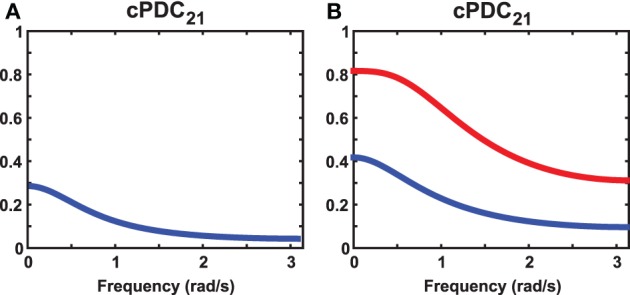
**Illustrative plots of the observations in Example 1. (A)** *cPDC*_21_ results for *e* = *f* = *g* = *h* = 0 in Example 1 revealing just one non-zero component under the *ad* = *bc* condition. **(B)** *cPDC*_21_ when *b* = *c* = 0 and non-zero *a* and *c* in Example 1 leading to two non-zero components for any non-zero values of the *e*, *f*, *g*, *h* coefficients (the graph shown was produced using *a* = 0.5, *b* = 0, *c* = 0, *d* = 1, *e* = 0.3, *f* = −0.1, *g* = 0.3, *h* = 0.4).

Even if *e*, *f*, *g*, *h* are non-zero, i.e., regardless of intrablock dynamics, *a* = *b* = 0 suffices to produce the single non-zero π^(*c*_1_)^_21_(ω) component (shown in Figure 2A) since block *X*_1_ interacts with block *X*_2_ exclusively through *Y*_4_, i.e., π^(*c*_2_)^_21_(ω) ≡ 0. In this case, since only *Y*_4_ is directly impacted by the interaction, only one combined source of variance exists even though two links exist between the blocks. Likewise if *b* = *d* = 0, even though two links leave *X*_1_, there is only one dynamical component that counts.

This contrasts with the situation when *b* = *c* = 0 where two non-zero π^(*c*_2_)^_21_(ω) coexist (Figure 2B) regardless of the values of *e*, *f*, *g*, *h* which, nonetheless, contribute to the relative size of the components.

**Figure 3:**
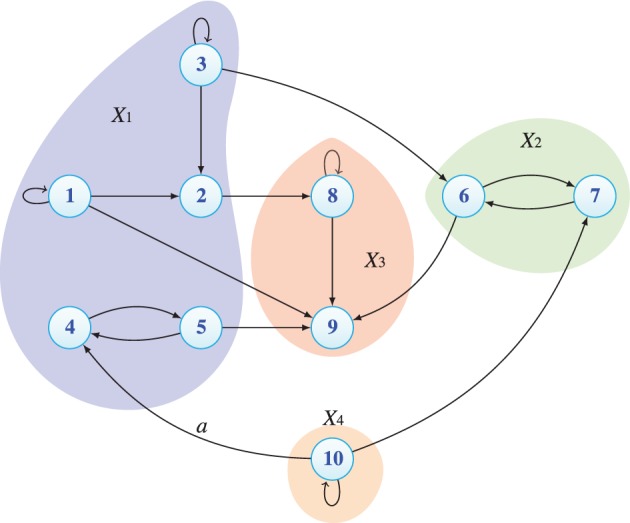
**Connectivity diagram for Example 2 portraying how the bock sets**. Note the effect of the value of the *a* parameter on cDC (Figure 5 versus Figure 6).

*Example 2*. In the next example, a 10-variate time series (*Y*_1_, …, *Y*_10_) follows the connectivity diagram represented in Figure 3. The multivariate time series is divided into four blocks (*X*_1_, *X*_2_, *X*_3_, and *X*_4_), where *X*_4_ only sends information and *X*_3_, which is an integrative block, only receives information. Block *X*_1_ has two functionally distinct internal parts, and only one is reached by outside influence. The scenario is fairly complicated and we next illustrate cPDC/cDC usefulness for understanding the underlying dynamic interaction between blocks.

To help interpret the results, we begin by describing the non-zero model coefficients and their dynamical effects. Observe that the model subscript indices in this example indicate the corresponding scalar process and not the block number.

1. Block *X*_1_ = [*Y*_1_ *Y*_2_ *Y*_3_ *Y*_4_ *Y*_5_]
  (29) $$\begin{array}{l} A_{1,1}(1)=1.98\;cos(\pi /50),A_{1,1}(2)=-{\left(.99\right)}^{2},\\ \;(\text{low frequency oscillator in }Y_{\text{1}}) \end{array}$$
  (30) $$A_{2,3}(1) =1,$$
  (31) $$\begin{array}{l} A_{3,3}(1)=1.98cos(\pi /2),A_{3,3}(2)=-{\left(.99\right)}^{2},\\ \;(\text{oscillator at midband }(\pi \text{/2})\text{in }Y_{\text{3}}) \end{array}$$
  (32) $$\begin{array}{l} A_{5,4}(1) = .99,A_{4,5}(1) = -.99,\\ \;(\text{oscillator at midband in }[Y_{\text{4}}\;Y_{\text{5}}]) \end{array}$$
  (33) $$A_{8,2}(1)=1,A_{8,2}(3)=1,\text{ (midband notch) }$$
  (34) $$A_{6,3}(1)=1,A_{6,3}(3)=1,\text{ (midband notch) }$$
  (35) $$A_{9,1}(1) =1,A_{9,5}(1) =1.$$
2. Block *X*_2_ = [*Y*_6_ *Y*_7_]
  (36) $$\begin{array}{l} A_{6,7}(1) = .99,A_{7,6}(1) = -.99,\\ \;(\text{oscillator identical to the }[Y_{\text{4}}\;Y_{\text{5}}]) \end{array}$$
  (37) $$A_{9,6}(1) =1.$$
3. Block *X*_3_ = [*Y*_8_ *Y*_9_]
  (38) $$A_{8,8}(1) = -1,A_{9,8}(1) = .5.$$
4. Block *X*_4_ = [*Y*_10_]
  (39) $$\begin{array}{l} A_{10,10}(1)=1.98\;cos(2\pi /3),A_{10,10}(2)=-{\left(.99\right)}^{2},\\ \;(\text{high frequency oscillator in }Y_{\text{10}}) \end{array}$$
  (40) $$A_{4,10}(1)=a,$$
  (41) $$A_{7,10}(1)=1.$$

**Figure 4:**
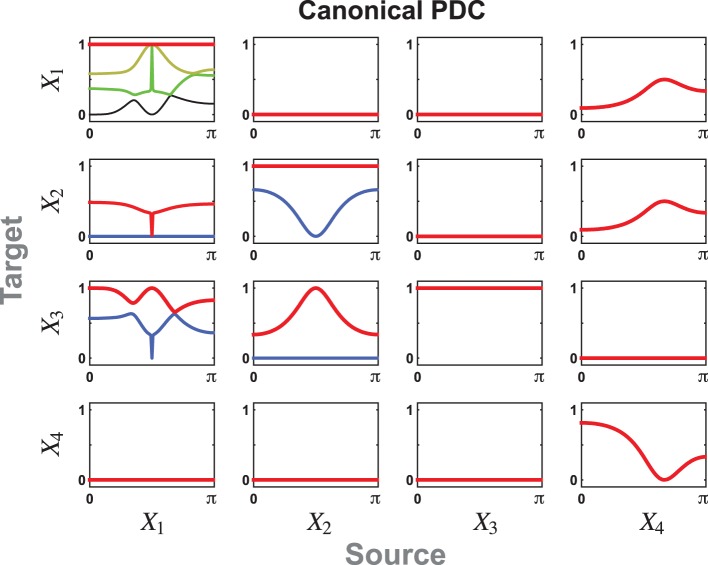
**The cPDC for Example 2 reflects the existence of the notch connecting filters from *X*_1_ to *X*_2_ and to *X*_3_**. The intrinsic dynamics of the oscillators from a subregion of *X*_1_ into *X*_3_ is apparent in the resonances of the largest cPDC component. The resonance within block *X*_2_ manifest itself in the single non-zero component into *X*_3_ while the effect of *X*_4_ reaches symmetrically into *X*_1_ and *X*_2_ via its single dynamic component. In this and following two figures, each subfigure may contain up to five cPDC/cDC components, given by *min*(*M_i_, M_j_*) as in Equations (22)/(23), represented in red, blue, yellow, green, or black lines in decreasing order of magnitude.

The resulting cPDC components can be appreciated in Figure 4 for |*a*| = 1. Among their interesting features is the existence of the notch filtered link from *X*_1_ to *X*_2_ and to *X*_3_ at midband. The effects of the low frequency dynamics due to *Y*_1_ and the midband resonance due to [*Y*_4_ and *Y*_5_] manifests itself as the strongest component from *X*_1_ to *X*_3_. Likewise the single link effect from *X*_2_ to *X*_3_ is readily apparent as the higher frequency resonances from *X*_4_ toward both *X*_1_ and *X*_2_. Both *X*_3_ components are identically equal to 1 since nothing leaves the block.

**Figure 5:**
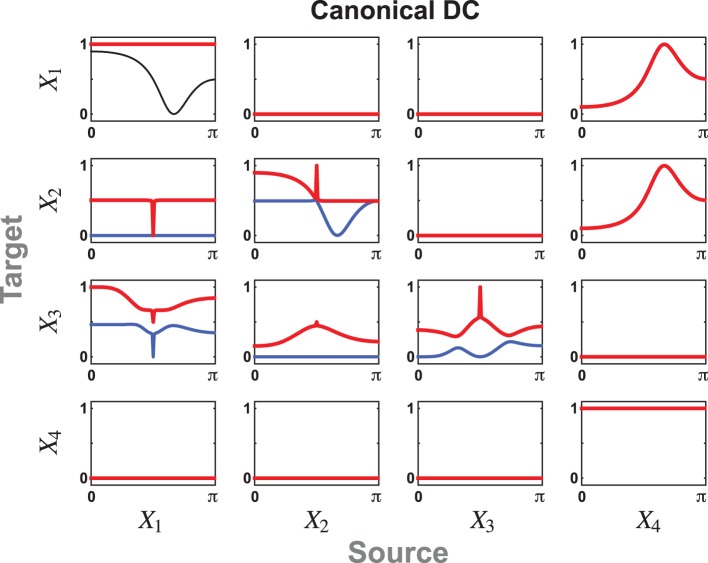
**cDC for Example 2 for *a* = −1 leading to a cancelation of the effect of *X*_4_ on *X*_3_ as the signal travels indirectly through two exactly identical structures but with opposite phases before reaching *X*_3_**. The notch filtering action is also apparent from the cDCs from *X*_1_ to *X*_2_ and *X*_3_.

**Figure 6:**
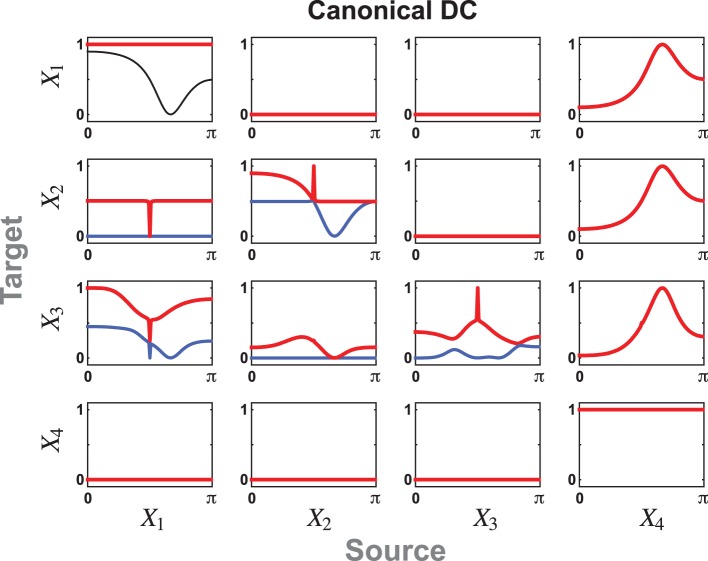
**cDC for Example 2 with *a* = 1 which differs from Figure 5 in the effect from *X*_4_ to *X*_3_ which no longer cancels out**.

The corresponding cDCs are portrayed in Figure 5 for *a* = −1 with no signal reachability from *X*_4_ to *X*_3_. This contrasts markedly with Figure 6 for *a* = 1 where *X*_4_'s indirect effects on *X*_3_ are not balanced out.

The effects of the notch connections are readily apparent in both cases. For example, the power associated with the notch frequencies are the local components to *X*_2_ and *X*_3_ and cannot be attributed to outside influence. For block *X*_1_ only one of the five components is different from 1 reflecting the contribution coming from *X*_4_.

### 5.2. Empirical data

**Figure 7:**
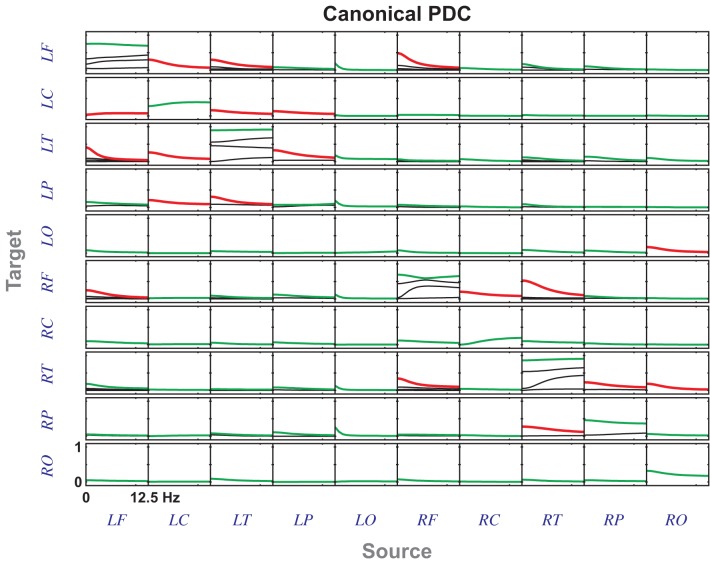
**cPDC from the Empirical Data example (section 5.2) from the left mesial ictal episode where the largest components are represented either in red or green**. cPDC values in red were arbitrarily considered significant and were pictorially summarized in Figure 8B.

**Figure 8:**
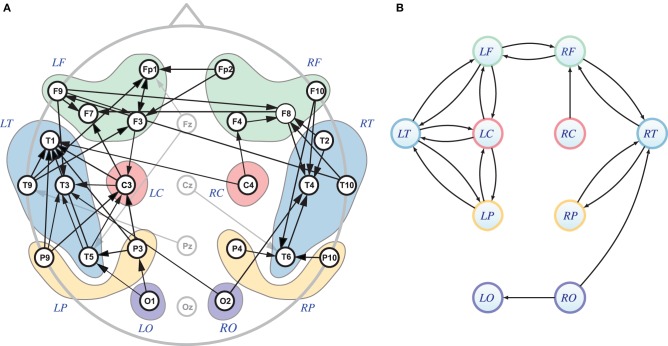
**(A)** This corresponds to Figure 7.13 from Sameshima et al. (2014) showing the gPDC connectivity graph (see arrows) and the scalp electrodes grouping sets corresponding to frontal (LF, RF), temporal (LT, RT), central (LC, RC), pariental (LP, RP), and occipital (LO, RO) areas. The midline electrodes in gray were not considered in this analysis. **(B)** Diagram for the significant first cPDC components in Figure 7 (red lines) showing scalp electrode set connections shown in **(A)**. Notice some divergences between gPDC and cPDC graphs possibly due the lack of proper rigorous statistics usage for cPDC significance level estimation, for instance, there is cPDC from RO to LO **(B)**, but gPDC O2 to O1 is absent **(A)**, while there is gPDC from C4 to T1 without corresponding cPDC from RC to LT.

This example is based on EEG data borrowed from Sameshima et al. (2014) (Ex. 7.7), which describes a left mesial temporal ictal episode monitored using an extended 10–20 system. The midline electrodes were excluded and left (L) and right (R) side electrodes were grouped as to whether they were frontal (F), central (C), parietal (P), temporal (T) or occipital (O) leading to the canonical PDCs portrayed in Figure 7 where the most important connecting blocks share a dominant low pass frequency canonical component of fairly identical shape pointing to the existence of a shared dominant connectivity dynamics behind the observation, see Figure 8A. Their connectivity is further summarized in Figure 8B.

## 6. Discussion

We showed that bPDC/bDC introduced in Takahashi (2009) and Faes and Nollo (2013) are block coherences between properly chosen vector time series. When the time series are Gaussian, this implies that bPDC/bDC represent mutual information rates between well defined underlying vector time series. This fully generalizes the results presented in Takahashi et al. (2010). To enhance the understanding of the possibly complex interaction between multiple time series and overcome some bPDC/bDC limitations, we showed that the latter can be decomposed in canonical terms that we call cPDC/cDC. These decompositions represent the various different modes of interaction whereby sets of time series interact. We introduced an explicit way to compute these new quantities and proved some of their properties. The usefulness of cPDC/cDC was illustrated by three examples.

### 6.1. bPDC and BDC as block coherences

Takahashi et al. (2010) showed that PDC from the *j*-th scalar time series to the *i*-th scalar time series is the coherence between the *i*-th innovation process and the *j*-th partialized process with a similar result for DC. It is natural to ask whether an analogous result holds for bPDC and bDC. We showed that this is indeed the case where bPDC/bDC represent block coherences relating subsets of adequately defined innovations/partialization processes (Takahashi, 2009; Nedungadi et al., 2011). At first sight these identities may seem surprising as both bPDC and bDC are fully multivariate and directional measures of dependence, whereas block coherences are at once block-pairwise and symmetric measures of dependence. Yet careful reading of Theorems 1 and 2 highlights that bPDC/bDC from *j* to *i* and bPDC/bDC from *i* to *j* are, in general, block coherences between distinct pairs of vector processes which explains their asymmetric nature and lends them their directed connectivity character. Also, we note that for both bPDC and bDC, the coherences involve innovation process subsets which explains their fully multivariate characteristic as measures. Another interesting observation is that since the innovation processes are uncorrelated to the past of the partialized processes by construction, in the case of bPDC only innovations in the past of the partialized process contribute to the coherence which explains why bPDC is a directed measure of dependence. An analogous observation holds for bDC. In the Gaussian case, the bPDC/bDC representation as a block coherence allows relating them to the mutual information rate between suitably chosen time series. Formally this justifies the idea that these quantities are *de facto* measures of information flow. For an interesting comparison between bPDC/bDC and Geweke's measure of linear feedback see Faes and Nollo (2013). As a small note for the reader, we observe that our definition of bPDC/bDC is slightly more general than the one proposed by Faes and Nollo (2013) because the covariance matrix of the innovations does not need to be diagonal as they assumed.

### 6.2. Canonical decomposition of directional measures

Given a pair of random vectors, it is natural to ask how to measure/represent dependence between them. In statistics, there are two main methods, both inspired by the basic Pearson correlation, to address this. The first one generalizes Pearson correlation directly using the determinants of the covariance matrix between and within each set of random variables. For time series, the equivalent measure in the frequency domain is the block coherence and the directed versions are bPDC and bDC. A second generalization rests on the idea of canonical correlation introduced by Hotelling (1936). There are several generalizations of canonical correlation for time series taylored specifically to infer Granger causality in the time domain (Sato et al., 2010; Wu et al., 2011), but, to the best of our knowledge, cPDC and cDC are the first proposals of canonical measures of directed dependence in the frequency domain.

One advantage of cPDC/cDC over bPDC/bDC is that canonical decomposition allows inferring the various different existing modes of interaction between sets of time series in close analogy to what is done for classical canonical correlation and principal component analyses. One should expect this to be useful when several signals are redundant, generated by similar mechanisms, or when there are several time series that do not significantly contribute to the interaction between sets of time series, e.g., when there are many brain areas that are not interacting with each other during some specific behavior. Besides, as we show in Theorem 4, we can recover the bPDC/bDC from the cPDC/cDC.

### 6.3. Interpreting cPDC/cDC

The main practical interest of cPDC/cDC is to allow the simplification of connectivity interpretations whilst giving new insights into the dynamical interaction between neural structures. We illustrated the achievable simplification using an EEG data set from an epileptic patient. We also showed how cPDC is related to the number of “modes” of interaction between sets of time series through the simple numerical Example 1 and via the slightly more complex Example 2. We expect that cPDC/cDC together with bPDC/bDC become useful tools for handling high dimensional data sets that are increasingly being recorded by several researchers.

We propose that a reasonable way to understand the usefulness of cPDC/cDC is to make an analogy with classical principal component and canonical correlation analyses. Therefore, similar heuristics could be applied in practical situations, for example, to decide the number of different components to include in the interpretation. The canonical time series *b*_*Y*_ and *b*_*Z*_ from section 4 (see also Brillinger, 1981) are analogous to the canonical variables from the classical canonical correlation analysis and can play a similar role for result interpretation.

Finally we remark that the computational procedures used for the present paper will be made available the PDC homepage at http://www.lcs.poli.usp.br/~baccala/pdc/canon together with the data used in section 5.2.

### Conflict of interest statement

The authors declare that the research was conducted in the absence of any commercial or financial relationships that could be construed as a potential conflict of interest.

## A. Appendix

### A.1. Proof of Theorems 1 and 2 and Corollaries 1 and 2

The proofs in this section follow the pattern of those in Takahashi et al. (2010). The chief difference lies in the care needed regarding the order of the products between the defining matrices. Here we exhibit the main proof ingredients for reader convenience, with further details available in Takahashi (2009) and Takahashi et al. (2010).

*Proof of Theorem 1 and Corollary 1*. Let *W* = [*Y^T^ Z^T^*]^*T*^ be a second order stationary process satisfying the boundedness condition, using the following well known identity for determinants (Lütkepohl, 1996)

(A1) $$\begin{array}{l} \det(S_{W}(\omega ))=\det(S_{ZZ}(\omega )\\ \;-S_{ZY}(\omega )S_{YY}^{-1}(\omega )S_{YZ}(\omega ))\det(S_{YY}(\omega )) \end{array}$$

leads to

(A2) $$\begin{array}{l} C_{YZ}^{\left(b\right)}(\omega )=1-\det(S_{ZZ}(\omega )\\ \;-S_{ZY}(\omega )S_{YY}^{-1}(\omega )S_{YZ}(\omega ))\det(S_{ZZ}^{-1}(\omega )) \end{array}$$

(A3) $$\begin{array}{l} =1-\det(S_{YY}(\omega )\\ \;-S_{YZ}(\omega )S_{ZZ}^{-1}(\omega )S_{ZY}(\omega ))\det(S_{YY}^{-1}(\omega )), \end{array}$$

under Equation (5).

Rewrite bPDC as

(A4) $$\begin{array}{l} \pi_{ij}^{\left(b\right)}(\omega )=1-\det(P_{jj}^{-1}(\omega )\\ \;-P_{jj}^{-1}(\omega ){\bar{A}}_{ij}^{*}(\omega )\Sigma_{ii}^{-1}{\bar{A}}_{ij}(\omega )P_{jj}^{-1}(\omega ))\det(P_{jj}(\omega )), \end{array}$$

so that using the following identities proved in Takahashi et al. (2010)

(A5) $$P_{jj}(\omega )=S_{\eta_{j}\eta_{j}}^{-1}(\omega ),$$

(A6) $$S_{\epsilon_{i}\eta_{j}}(\omega )={\bar{A}}_{ij}(\omega )S_{\eta_{j}\eta_{j}}(\omega ),$$

and for all ω ∈ [−π, π)

(A7) $$S_{\epsilon_{i}\epsilon_{i}}(\omega )=\Sigma_{ii},$$

back substituted into Equation (A4) leads to

(A8) $$\begin{array}{l} \pi_{ij}^{\left(b\right)}(\omega )=1-\det(S_{\eta_{j}\eta_{j}}(\omega )\\ \;-S_{\eta_{j}\epsilon_{i}}(\omega )\Sigma_{ii}^{-1}S_{\epsilon_{i}\eta_{j}}(\omega ))\det(S_{\eta_{j}\eta_{j}}^{-1}(\omega )), \end{array}$$

so that using Equation (A2) shows that the right-hand side of Equation (A8) actually is *C*^(b)^_ϵ_*i*_η_*j*__(ω) as we set out to prove. Corollary 1 is immediate from Theorem 1 and Equation (7).                          □

*Proof of Theorem 2 and Corollary 2*. Theorem 2 is obtained by rewriting *C*^(*b*)^*_X_i_ζ_j__*(ω) using Equation (A3) noting that

(A9) $$S_{X_{i}\zeta_{j}}(\omega )={\bar{H}}_{ij}(\omega )S_{\zeta_{j}\zeta_{j}}(\omega )$$

and for all ω ∈ [−π, π)

(A10) $$S_{\zeta_{j}\zeta_{j}}(\omega )=\Theta_{jj}^{-1}.$$

Corollary 2 follows from Theorem 2 and Equation (7).                          □

### A.2. Proof of theorems 3 and 4

Brillinger, (1981, chapter 10) introduced the idea of canonical coherence for time series. We restate his result under our notation as the following theorem.

**Theorem 5** (Brillinger, Theorem 10.3.2) *Let X and Y be m_1_ and m_2_-dimensional time-series jointly satisfying the boundedness condition. For m* ≤ min{*m*_1_, *m*_2_}, *the following identity holds:*

(A11) $$C_{XY}^{\left(c_{m}\right)}(\omega )=\lambda^{m}(S_{YY}^{-1}(\omega )S_{YX}(\omega )S_{XX}^{-1}(\omega )S_{XY}(\omega ))$$

(A12) $$=\lambda^{m}(S_{XX}^{-1}(\omega )S_{XY}(\omega )S_{YY}^{-1}(\omega )S_{YX}(\omega )).$$

*Proof of Theorem 3*. From Equations (18), (A11), we have

(13) $$C_{\epsilon_{i}\eta_{j}}^{\left(c_{m}\right)}(\omega )=\lambda^{m}(S_{\eta_{j}\eta_{j}}^{-1}(\omega )S_{\eta_{j}\epsilon_{i}}(\omega )S_{\epsilon_{i}\epsilon_{i}}^{-1}(\omega )S_{\epsilon_{i}\eta_{j}}(\omega )).$$

Now, from Equations (A5), (A6), and (A7) it follows that

(A14) $$S_{\eta_{j}\eta_{j}}^{-1}(\omega )S_{\eta_{j}\epsilon_{i}}(\omega )S_{\epsilon_{i}\epsilon_{i}}^{-1}(\omega )S_{\epsilon_{i}\eta_{j}}(\omega )={\bar{A}}_{ij}^{*}(\omega )\Sigma_{ii}^{-1}{\bar{A}}_{ij}(\omega )P_{jj}^{-1}(\omega ),$$

which proves Equation (20).

To prove Equation (21), we use Equations (19), (A12) to obtain

(A15) $$C_{X_{i}\zeta_{j}}^{\left(c_{m}\right)}(\omega )=\lambda^{m}(S_{X_{i}X_{i}}^{-1}(\omega )S_{X_{i}\zeta_{j}}(\omega )S_{\zeta_{j}\zeta_{j}}^{-1}(\omega )S_{\zeta_{j}X_{i}}(\omega )).$$

Finally, from Equations (A9), (A10), we have

(A16) $$S_{X_{i}X_{i}}^{-1}(\omega )S_{X_{i}\zeta_{j}}(\omega )S_{\zeta_{j}\zeta_{j}}^{-1}(\omega )S_{\zeta_{j}X_{i}}(\omega )=S_{ii}^{-1}(\omega ){\bar{H}}_{ij}(\omega )\Theta_{jj}^{-1}{\bar{H}}_{ij}^{*}(\omega ),$$

which concludes the proof.                          □

*Proof of Theorem 4*. Rewrite bPDC as

(A17) $$1-\pi_{ij}^{\left(b\right)}(\omega )=\det(I-{\bar{A}}_{ij}^{*}(\omega )\Sigma_{ii}^{-1}{\bar{A}}_{ij}(\omega )P_{jj}^{-1}(\omega )).$$

Now, Equation (22) is a straightforward consequence of the relationship between eigenvalues and the determinant of a matrix. A similar argument proves Equation (23).                          □
